# Draft genome sequence of *Acerihabitans* sp. strain TG2^T^, isolated from an Arctic tundra soil sample

**DOI:** 10.1128/mra.00024-24

**Published:** 2024-04-15

**Authors:** V. A. Shcherbakova, A. G. Zakharyuk, V. E. Trubitsyn

**Affiliations:** 1 Skryabin Institute of Biochemistry and Physiology of Microorganisms, Pushchino Scientific Center for Biological Research of the Russian Academy of Sciences, Pushchino, Moscow Oblast, Russia; California State University San Marcos, San Marcos, California, USA

**Keywords:** genome analysis, Arctica, psychrophiles

## Abstract

*Acerihabitans* sp. type strain TG2^T^ (VKM B-3773^T^) is a gram-negative, anaerobic psychrophilic bacterium that was isolated from a tundra soil sample selected by the Bykovsky Peninsula (Russia). This report describes the generation and annotation of the 5.3 Mb draft genome sequence of *Acerihabitans* sp. strain TG2^T^.

## ANNOUNCEMENT

An anaerobic psychrophilic bacterium, *Acerihabitans* sp. type strain TG2^T^ (VKM B-3773^T^), was isolated from the upper horizon of a tundra soil sample (depth 8–17 cm) collected by the western part of the Ivashkina lagoon by the Bykovsky Peninsula, Russia in 2021 (71.72N 129.28E). Strain isolation was performed according to the Hungate anaerobic method (1) by the dilution method on medium (2) with xylose as the substrate. Colonies were obtained by the “roll-tube” method using 2% (wt/vol) agar medium. Cells of the strain were gram-negative, motile, non-sporulating short rods. Strain TG2^T^ grew in the temperature range from 0 to 25°C (optimum 8°C) and at pH 6.0–8.0 (optimum pH 7.5).

The strain was grown from a single colony. Colonies for DNA isolation and sequencing were grown on a solid medium at 8°C and pH 7.5 for 4 weeks. The Hungate tube with colonies was transferred to the BioSpark Company (Troitsk, Russia) for genomic DNA preparation and sequencing. Genomic DNA was isolated with the FastDNA spin kit (MP Biomedicals, USA) by the column method with deposition on silica gel. The libraries were synthesized using KAPA HyperPlus kits (Kapa Biosystems, USA) in accordance with the manufacturer’s recommendations. Sequencing was performed on the Illumina NovaSeq 6000 platform, and a paired-end library with a total of 2,786,968 read pairs and a read length of 2 × 150 bp was obtained. The Kbase (3) online platform with associated tools was used for genome assembly and analysis. The quality of the reads was assessed using the FastQC v.0.12.1 program (4). Reads were processed in the Trimmomatic (5) program v. 0.36 with the parameters “HEADCROP = 15, MINLEN = 50” and removing adapters from the TruSeq3-PE-2 set. The assembly with the best parameters was obtained using the Unicycler v.0.4.8 assembler (6). Genome completeness and contamination were evaluated using CheckMv.1.0.18 (7). Taxonomy was assigned using GTDBtk v.1.7.0 (8) and TYGS server (9). The orthoANI calculation was made using the online service https://www.ezbiocloud.net/tools/ani. Alignment of reads to genomes, determination of coverage, and the number of aligned reads were carried out using the bowtie2 v. 2.3.2 program (10).

The genome was annotated using the NCBI Prokaryotic Genome Annotation Pipeline (PGAP) (11). The genome contained 108 contigs with a maximum length of 420,954 bp. The assembled draft genome sequence length was 5,292,910 bp. The G + C content was 51.1%, the scaffold N_50_ value was 150.9 kb, and the L_50_ value was 12. Reads alignment demonstrated that the genome accounted for 68.33% of the reads, with coverage of 106.34 ± 40.32. Genome completeness was 100%, contamination was 0.96%.

The total number of genes was 4,950, including 4,734 protein-coding sequences, 61 tRNA, and 2 rRNA genes (one 16S and one 23S). The closest related type strain, according to TYGS, was *Acerihabitans arboris* SAP-6^T^ (assembly GCA_010131535.1, WGS WUBS01), isolated from tree sap (Jeju, South Korea) (12) with DNA-DNA hybridization (DDH) 22.6% and orthoANI 78.2%. 16S rRNA gene was extracted from the genome. The similarity between nucleotide sequences of 16S rRNA genes of strains TG2^T^ (1551 bp length) and SAP-6^T^ (MN737198.1) was 98.2%. Taken together with previous data, this may indicate that the studied strain is a representative of a new species of the genus *Acerihabitans*.

## Data Availability

The raw sequencing reads were deposited in the Sequence Read Archive (SRA) (SRA accession numbers SRR26888815 and SRR26888816). The genome sequence of *Acerihabitans* sp. strain TG2T is available in GenBank under accession number JAYGJO000000000.1 (BioProject accession number PRJNA1041642 and BioSample accession number SAMN38289666), and the 16S rRNA nucleotide sequence was deposited under accession number PP024248.
